# Nervous Debility

**Published:** 1895-05

**Authors:** Sarah N. Smith

**Affiliations:** New York


### NERVOUS DEBILITY.

SARAH N. SMITH, M. D., NEW YORK.

October 31st, 1894, I was called to a Mrs. ——, a lady something over thirty perhaps, who had suffered from a neurosis fourteen months or more. She had one child, a boy four years of age. Like the one in Scripture, "She had suffered many things, from many physicians," was nothing better, but rather grew worse. This case had baffled the skill of five physicians, each in his turn. I say skill, because they must have been skillful, of course, as they were from the "regular school," and men doctors at that. Notwithstanding, each in his turn failed to benefit the subject of this paper.

This patient was a lady of culture and refinement, reared tenderly by her parents, and tenderly cared for by a kind, indulgent husband, who had come to feel a fear that she never would be well again.

On my first visit the patient was suffering from an acute attack of indigestion, accompanied with a diarrhœa, that necessitated a limited and judicious diet, as she was already weak and exhausted, with stomach and bowels aggravated by the least amount of food, so that nausea and vomiting would appear at the slightest provocation.

I made a careful examination of the patient; found her weak and feeble, in body and mind, without any hope of recovery. I saw nothing that appeared to be incurable, and I felt that with close care and attention she could be restored to fair health at least. I reported to her friends accordingly, which report they received, I thought, with much incredulity. But that did not cause me to waver in the least, as I knew too well what Homœopathy could do for such cases.

The first object to be gained was to put the stomach in working order, and stop this drain upon the system, which was already very much depleted. The symptoms present indicated Arsenicum. I left Dunham's 200 to be given in water every two hours until my next visit. I called at 10 A. M. the next day; I found her greatly improved by sleep and rest, and the trouble from the bowels mostly removed.

She was very sure that she never should be any better—had nothing to live for only to suffer. "Wouldn't you like to live to see your little boy grow to manhood and be a blessing to his parents and others?"

I spoke of the child to see if I couldn't arouse some interest in her, as she was very indifferent to all her surroundings; perfectly apathetic about everything. She complained of very strange, or as she expressed it, "queer" feelings in the head most of the time—indescribable, she said. She said her feet were cold, night and day, requiring a hot-water bag most of the time, showing the circulation to be very poor, liver torpid, stomach weak, and indigestion as a natural result.

For this condition I gave Lycopodium; from Dunham's 200 to Fincke's 50M. This remedy did good service in many ways. The patient couldn't see any improvement, and felt sure that her bad feelings would return as they always had. But Similia similibus applied, according to the law of Hahnemann, the Great High Priest of Homœopathy, works to cure, and it did not fail me in this case.

But the more difficult and important features in this case were yet to be dealt with: her mental condition.

She was not inclined to make the least effort of any kind; quite satisfied to lie in bed undisturbed without care or thought, similar to Apis, but her great depression, with weeping and melancholy and dread of some great misfortune, gave preference to Calcarea-carb., which is also a lazy fellow—then she had not the jealousy of Apis. I gave Dunham's 200, which gave relief and quiet sleep, but the result was not satisfactory, to me at least. I then carefully reviewed the conditions to make sure that I had selected the indicated remedy. This drug seemed to me to cover the totality of her symptoms. I thought best, however, to change the potency, and gave the patient one powder of Descheres' CM, with "Saccharum-lactis."

When I went to her the next morning, I had no need to inquire how she felt, as her whole make-up showed the improvement. She couldn't help but look pleasant (laugh ye who will). Is infinity limited or measured by any boundary lines? Nay, verily. The human body is composed of millions of cells, so minute as to be nearly invisible with the highest power of the microscope, hence requires minute portions of drugs to affect them. The infinitesimal dose as here used.

The patient was quite unwilling to say that she felt any better, but admitted that she didn't feel as blue and so much like crying all the time.

I felt that we had gained a great victory, as I would give more for a thimbleful of mental symptoms, improved, than for the relief of a bushel of bodily aches and pains, common to most sick people.

This patient was not free from pain, by any means, but I knew that would be made right, when we could counteract the effect of the drugs that she had taken all these months. Her stomach had been converted into an apothecary shop, and it rebelled, as it didn't like the drug business.

This necessitated a change in the treatment, and with this change health and comfort were near at hand. But it was not all joyous for the doctor and attendants even now, for there was a vast amount of imaginative ailments with this patient, as there is with most cases of nervous debility, depending, of course, something upon the cause of the debility. These symptoms gradually yielded to the indicated remedies, until all doubts of her recovery had disappeared, and the patient quite herself again, in most respects. She manifested more interest in her surroundings and was more hopeful for the future, and planned for the same.

It was the first experience of this family with Homœopathy, and it was not strange if they felt shaky as to the results.

My faith, however, was sufficient for us all. The Good Book says, "According to your faith be it unto you." This case was no exception. She continued to improve, went daily for a walk accompanied by her nurse. She was not anxious to walk, but was willing to do what would improve her most and restore her health.

I visited her daily for a month until I left the city two days for Thanksgiving. When I returned on Saturday, she was feeling very well and going down to take some of her meals with her family.

I visited her frequently through December and left her at that time comparatively well. Her husband wrote me January 7th, that she continued to improve and was doing well.

At that time I was taken ill and confined to my bed for nearly a month and did not see her again.

This case shows the superiority of Homœopathy over Allopathy. She was under treatment of the latter some fourteen months not to improve, but actually to grow worse, until her friends despaired of her ever being well again. Two months of pure Homœopathy, applied as the Great Master teaches, did the work, and placed the patient on a living basis once more.

